# A priori Prediction of Neoadjuvant Chemotherapy Response and Survival in Breast Cancer Patients using Quantitative Ultrasound

**DOI:** 10.1038/srep45733

**Published:** 2017-04-12

**Authors:** Hadi Tadayyon, Lakshmanan Sannachi, Mehrdad J. Gangeh, Christina Kim, Sonal Ghandi, Maureen Trudeau, Kathleen Pritchard, William T. Tran, Elzbieta Slodkowska, Ali Sadeghi-Naini, Gregory J. Czarnota

**Affiliations:** 1 Physical Sciences, Sunnybrook Research Institute, Sunnybrook Health Sciences Centre, Toronto, ON, Canada; 2 Department of Medical Biophysics, Faculty of Medicine, University of Toronto, Toronto, ON, Canada; 3 Department of Radiation Oncology, Odette Cancer Centre, Sunnybrook Health Sciences Centre, Toronto, ON, Canada; 4 Division of Medical Oncology, Department of Medicine, Sunnybrook Health Sciences Centre, Toronto, ON, Canada; 5 Department of Anatomic Pathology, Sunnybrook Health Sciences Centre, Toronto, ON, Canada; 6 Department of Radiation Oncology, Faculty of Medicine, University of Toronto, Toronto, ON, Canada

## Abstract

Quantitative ultrasound (QUS) can probe tissue structure and analyze tumour characteristics. Using a 6-MHz ultrasound system, radiofrequency data were acquired from 56 locally advanced breast cancer patients prior to their neoadjuvant chemotherapy (NAC) and QUS texture features were computed from regions of interest in tumour cores and their margins as potential predictive and prognostic indicators. Breast tumour molecular features were also collected and used for analysis. A multiparametric QUS model was constructed, which demonstrated a response prediction accuracy of 88% and ability to predict patient 5-year survival rates (p = 0.01). QUS features demonstrated superior performance in comparison to molecular markers and the combination of QUS and molecular markers did not improve response prediction. This study demonstrates, for the first time, that non-invasive QUS features in the core and margin of breast tumours can indicate breast cancer response to neoadjuvant chemotherapy (NAC) and predict five-year recurrence-free survival.

Locally advanced breast cancer (LABC) is an aggressive subtype of breast cancer which is clinically defined as a tumour that is greater than 5 cm or involves the skin or chest wall. LABC also includes inflammatory breast cancers and patients with fixed axillary lymph nodes or ipsilateral supraclavicular, infraclavicular, or internal mammary nodal involvement^1^. Neoadjuvant chemotherapy (NAC) is increasingly becoming the modality of choice in the upfront treatment of LABC patients with the availability of a wide spectrum of systemic and targeted drugs. However, only 10–20% of patients achieve pathologic complete response to NAC^1^, which is determined at the end of the several-month treatment. Thus, the introduction of a biomarker predictive of tumour response to NAC prior to treatment could facilitate personalized treatment, resulting in improved tumour response to NAC and a better long-term outcome. From a biological perspective, immunohistochemical markers such as Ki-67 and human epithelial growth factor receptor 2 (HER2) and circulating tumour nucleosomes have been suggested to be predictive of the likelihood of breast tumour response to NAC prior to treatment^2^,^3^,^4^,^5^. From an imaging perspective, a recent study involving diffuse optical spectroscopic tomographic (DOST) imaging of LABC patients indicated that pathologically complete response patients have significantly higher hemoglobin concentration levels than those with pathologically incomplete response with p = 0.01^6^. There are studies of other methods including magnetic resonance imaging (MRI), QUS, and DOST as imaging methods^7^ and circulating DNA and RNA-integrity measurements^8^ for response prediction but only after chemotherapy has been started. Despite initially convincing results, those previous studies have not yet borne out on larger cross-validated patient populations.

Clinical ultrasound is widely used in medicine due to its cost-effectiveness, large penetration depth (~ 7 cm) and real-time imaging capability. Furthermore, the radiofrequency (RF) backscatter signal derived from ultrasound provides information about the tissue microstructure otherwise not resolvable by conventional ultrasound images (B-mode images). Quantitative ultrasound (QUS) techniques examine the frequency dependence of the RF signal backscattered from tissues and have been applied *in vivo* in a variety of applications to reveal information about tissue microstructure, enabling the differentiation of disease from normal tissue and the characterization of disease into its subtypes. For instance, parameters derived from linear regression analyses of RF power spectra, including midband fit (MBF), spectral slope (SS), and spectral 0-MHz intercept (SI), have been used to characterize intraocular tumours and to detect prostate cancer, cardiovascular disease, and cancerous lymph nodes^9^,^10^,^11^,^12^. Broader frequency bandwidths further permit estimation of advanced parameters such as average (effective) scatterer diameter (ASD) and average (effective) acoustic concentration (AAC), which are derived by fitting a scattering model to the RF data^13^. These parameters have been demonstrated to be effective in studies differentiating mouse carcinomas from rat fibroadenomas^14^ and have demonstrated potential for use in breast tumour grading^15^,^16^. Recent pre-clinical studies have demonstrated using high frequency ( > 20 MHz) and clinical frequency ( < 10 MHz) ranges of ultrasound that QUS can be used to detect and quantify tumour cell death *in vivo* in response to various treatments including photodynamic therapy, radiation therapy, chemotherapy, and anti-vascular therapy^17^,^18^,^19^,^20^. Furthermore, a recent pilot clinical study by Sadeghi-niani *et al*.^21^ demonstrated the effectiveness of the textural features of QUS spectral images of MBF and SI in the detection of patients’ breast tumour responses to neoadjuvant chemotherapy as early as one week into their several-month treatment. Similarly, Sannachi *et al*.^22^ demonstrated that the mean of intensity of ASD and AAC images derived from US backscatter are effective in a similar clinical application. Furthermore, Tadayyon *et al*.^23^ demonstrated that a QUS multi-parametric imaging approach utilizing MBF, SS, and mean scatterer spacing parameters submitted to a k-nearest neighbor (k-NN) classifier is an effective method of predicting breast tumour responses to chemotherapy early during the course of the treatment. However, to date, all reported QUS analyses with regards to response prediction have been limited to the tumor core region, neglecting the tissue immediately surrounding the tumor that may include microscopic tumor extensions. There is mounting clinical evidence of increased risk of local recurrence of the breast tumor if the tumor margin is found to be positive under the microscope after the breast conserving surgery^24^. In order to minimize the chances of positive margin (i.e. residual malignant tumor cells found at the margin of the excised tumor bed), generally 3–5 mm of normal breast tissue around the tumor bed is removed during breast conserving surgery^24^. Thus, including this “rim” of surrounding tissue in the image analysis may reveal more information about the tumor and better characterize its chemotherapy responsiveness.

Using the same database of patients and their breast ultrasound images, the study here builds upon previous studies by Sadeghi-Naini *et al*.^21^, Sannachi *et al*.^22^, and Tadayyon *et al*.^23^ with the following key contributions:

1. Baseline QUS imaging features were evaluated in terms of their ability to predict breast tumour response in advance of any treatment.
2. In addition to examining textural features within the tumor core as done previously, QUS features in the tumor’s surrounding tissue, referred to here as the margin, extending 3–10 mm from the core were analyzed. Quantitative features such as core-to-margin ratio (CMR) and core-to-margin contrast ratio (CMCR) QUS features were examined here, for the first time, and were considered as potential ultrasound-based biomarkers of tumour aggressiveness that determine the likelihood of tumour response.
3. Molecular biomarkers including estrogen receptor (ER), progesterone receptor (PR), and HER2 were investigated as stand-alone and as additional biomarkers for response prediction.
4. In addition to good versus poor response classification done previously in a different therapy monitoring study^23^, complete versus incomplete response classification was also investigated but for data obtained a priori to patient treatment.

Linear regression spectral parameters including MBF, SS, and SI, and more complex backscatter model parameters including ASD and AAC were computed from a region of interest (ROI) within the tumour and its margin. Additionally, an attenuation coefficient estimate (ACE) was obtained from the core ROI as a spectral correction factor and as a potentially predictive parameter. Higher-order statistical (or textural) and image quality features were extracted from parametric images based on the aforementioned parameters and were subsequently submitted to a classifier to predict the response of the patients. Furthermore, molecular markers including ER, PR, and HER2 were investigated as separate and additional predictors of response. Three types of classifiers were investigated for response prediction: Fisher’s linear discriminant (FLD), k-NN, and support vector machine (SVM). The clinical and pathological response (good versus poor response and complete versus incomplete response) of each patient was determined at the end of the treatment according to response evaluation criteria in solid tumours (RECIST)^25^ and was used to evaluate the classification sensitivity, specificity, accuracy, and area under the ROC curve (AUC). Lastly, Kaplan-Meier survival analysis was performed in order to determine, retrospectively, the linkage of QUS methods to predicting the recurrence-free survival (RFS) of LABC patients. The results here are supported by sensitivity, specificity, accuracy, and AUC measurements obtained using leave-one-out cross-validation.

## Materials and Methods

The prospective study was conducted under the regulations and guidelines in accordance with the research ethics board at Sunnybrook Health Sciences Centre (SHSC), Toronto Canada. All experimental protocols were reviewed and approved by the research ethics board at SHSC prior to commencing the study. After obtaining informed consent, ultrasound radiofrequency (RF) data and corresponding anatomical images (B-mode) were acquired from the affected breast of 56 LABC patients using a clinical ultrasound system equipped with a 6 MHz center frequency linear array transducer (Sonix RP system, L14-5/60, Ultrasonix, Vancouver, Canada). All patients enrolled in this study were diagnosed with LABC with the exception of four early stage breast cancer patients who received NAC due to presenting with lymph node disease, and long surgery wait time. There were no cases of inflammatory breast cancer in this study. All patients completed a full course of NAC, which lasted 4–6 months, after which they underwent mastectomy or lumpectomy. The imaging session occurred within one week of diagnosis and prior to NAC initiation. Ultrasound RF data were acquired at a sampling rate of 40 MHz. The focus was set at the midline of the tumour using electronic beam focusing, and the imaging depths varied from 4 to 6 cm, depending on tumour size and location. Approximately 4–7 image planes were acquired, covering the full extent of the tumour volume, spaced at 5 mm intervals. In order to capture the heterogeneity of the tumour, ROIs were selected from central and peripheral zones of the tumour symmetrically.

### Patient clinical characteristics

Patient clinical features including age, tumour size, hormone receptor statuses, and RECIST-based tumour size change were obtained from clinical evaluations and reports, biopsy results, and imaging reports. All patients in this study completed full courses of chemotherapy. Over 90% of patients received anthracycline-taxane based regimens. Enrolment of patients in terms of chemotherapy was not limited to one tumour type or particular regimen in order to facilitate maximal enrolment. Patients had clinical MRI scans before treatment and after as part of standard of care practices for tumour size measurements and assessment of chest wall involvement. The RECIST-based change was defined as the percent change in tumour size (longest diameter) between pre-treatment and post-treatment times (several months later). Pre-/post-treatment tumour sizes were obtained from pre-/post-treatment magnetic resonance imaging and post-surgical pathology reports. After mastectomy/lumpectomy, standard clinical pathology was carried out as part of patients’ standard of care. The method of clinical and pathological response determination (good/poor) in this study was based on the RECIST criteria with the addition of accounting for residual tumour cellularity upon completion of chemotherapy. A recent study demonstrated that residual tumour cellularity is also an important prognostic factor in breast cancer neoadjuvant treatment, which should be taken into account in conjunction with the RECIST metric^26^. Accordingly in this study, a patient was deemed to be a good response patient if the sum of the lengths of the tumor foci was reduced by more than 30% or if in the non-mass enhancing area, the pathologically determined residual tumor cellularity was low. Conversely, a patient was considered a poor response patient if the sum of the lengths of their tumor foci was reduced by less than 30% or the residual tumor cellularity remained high. In cases (infrequent) where the RECIST-based response conflicted with the pathological response, the pathological response was used to determine the true response. For this study, histopathologic measures of response were also calculated using cellularity, based on methods described by Miller & Payne (MP)^27^. Response (good/poor) was also evaluated from MP, using a threshold of 3, where patients with a Miller-Payne score 3 or greater were labelled as good response patients, and the remaining were labelled as poor response patients (MP scores are based on categorical data ranging from 1 to 5; where 1 corresponds to no changes in tumour cellularity and 5 corresponds to complete disappearance of invasive tumour cells, relative to biopsy). Alternatively, tumour response can be classified as either a complete response or incomplete response, where a complete response is indicated by complete disappearance of residual disease upon post-surgical pathology examination and incomplete response is indicated as otherwise. Detailed clinical characteristics of patients involved in this study, including patient age, pre-treatment tumour size, pathology, hormone receptor overexpression, as well as treatment type are presented in Supplementary Table 1. Clinical outcome of patients, including residual tumour size, pathology notes, and clinical and pathological response in terms of good response versus poor response, complete response versus incomplete response, and MP-scores are presented in Supplementary Table 2. Oncotype Dx and tumour infiltrating lymphocytes were not evaluated since the former is not used in the Canadian clinical setting with NAC, and the latter is not standard clinically available information. Recurrence-free survival was determined using any recurrence (local or regional [including invasive ipsilateral tumour and invasive locoregional tumour], or distant) or death due to any cause (both breast cancer and non-breast cancer causes of death).

### Quantitative ultrasound parameter evaluation

**Figure 1:**
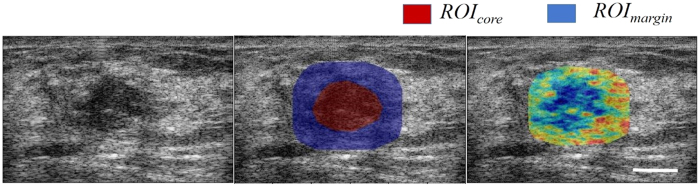
Representative ultrasound B-mode (left), corresponding ROIs with a 5 mm margin thickness (center), corresponding SI parametric image (right). Scale bar: 1 cm.

For QUS analysis purposes, an ROI was generated by manually outlining the core of the tumour (the tumours are locally advanced and are readily detectible). This was referred to as *ROI*_*core*_. In order to compare the QUS features inside the tumour with those immediately surrounding the tumour where there may be microscopic tumour extensions, another ROI was generated automatically, which was referred to as *ROI*_*margin*_. This ROI was prescribed with a pre-defined thickness of 3 mm, 5 mm, or 10 mm. An example tumour segmentation with a 5 mm margin is presented in Fig. 1. The core/margin ROI pairs were selected from 4–7 image planes containing the tumour, from which QUS parameters were evaluated and averaged. Each ROI was divided into analysis blocks of size 2 × 2 mm with 80% adjacent overlap in axial and lateral directions. This ensured a standardization of the QUS analysis block size among all patients. A normalized power spectrum was computed from each analysis block using a reference phantom^15^, and then corrected for frequency-dependent attenuation that was estimated locally^16^. From each analysis block, spectral parameters including MBF, SS, SI and backscatter model parameters including ASD and AAC were computed using the methods described in Tadayyon *et al*.^15^ which were originally developed by Lizzi *et al*.^28^ and Insana *et al*.^13^, respectively. By generating a spatial map of the parameter values computed over all analysis blocks, a color-coded image was generated, referred to as a parametric image. In order to illustrate heterogeneity, a representative conventional ultrasound image (B-mode image) of a breast tumour, its corresponding core and margin ROIs, and a resulting SI parametric image are presented in Fig. 1. Additionally, an attenuation coefficient estimate (ACE) was computed using the spectral difference method^29^. The ACE was a single value estimated from *ROI*_*core*_, and thus, no parametric image was available for this parameter. In order to characterize structural patterns in *ROI*_*core*_, a gray-level co-occurrence matrix (GLCM)–based texture analysis was performed on the newly obtained parametric images as described in Tadayyon^b^ *et al*.^16^ which was initially developed by Haralick *et al*.^30^. The GLCM is a matrix that represents the angular relationship between neighboring pixels as well as the distance between them^30^. The following four textural features were extracted from each ROI: contrast (CON), correlation (COR), energy (ENE), and homogeneity (HOM) as per Haralick *et al*.^30^. Signal-to-noise ratio (SNR) is an image quality metric which compares the level of desired signal to the level of background noise. Contrast-to-noise ratio (CNR) is similar to SNR but also considers bias in an image. Measures of SNR and CNR are commonly used to evaluate image quality when modifying an existing imaging system to improve image quality, such as in cone-beam computed tomography^31^. In this study, two image quality features were defined in a manner similar to SNR and CNR in ref. 31, with the aim to compare pixel intensities between two ROIs. The core-to-margin ratio (CMR) and core-to-margin contrast ratio (CMCR) were defined as follows:


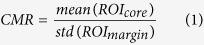



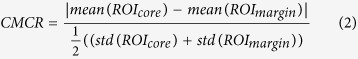


Supplementary Table 3 presents the parametric image features investigated and the locations where they were computed in a categorized manner: first order statistics (mean), second order statistics (CON, COR, ENE, and HOM), and image quality (CMR and CMCR).

### Tissue classification and statistical analyses

Prior to performing patient response classification based on their tumour QUS and/or molecular features, the features were sorted by their statistical significance, from lowest to highest p-value (depending on data normality, unpaired t-test or Mann-Whitney test, one-tail, α=0.05). Starting with the lowest p value feature as the initial model, features were sequentially added to or discarded from the model until there was no improvement in classification accuracy based on RECIST and pathological response. This process is referred to as sequential forward feature selection^32^. The set of features of the final model obtained using sequential forward feature selection is referred to here as the optimal feature set. Three types of classifiers were used for comparison – FLD, SVM, and k-NN. Comparisons were made based on classification sensitivity, specificity, accuracy, and AUC. For good versus poor response classification, sensitivity was defined as the ratio of the number of true good response patients to the total number of good response patients (expressed as a percentage) and specificity was defined as the ratio of the number of true poor response patients to the total number of poor response patients in percentage. For complete versus incomplete response classification, sensitivity was defined as the ratio of the number of true complete response patients to the total number of complete response patients in percentage. Specificity was defined as the ratio of the number of true incomplete response patients to the total number of incomplete response patients in percentage. Accuracy was determined as the percentage of total number of correctly classified patients to the total number of patients. The classification scores were bootstrapped 1000 times in order to obtain the 95% confidence intervals of the AUC. All statistical and machine learning analyses including bootstrapping were implemented using MATLAB R2011B (Mathworks, USA). An FLD is a linear classifier which projects the multidimensional data onto a feature space which maximizes the ratio of between-class to within-class variance and performs well when the data can be separated by a line. An SVM builds a model (from the training data) so as to have the largest gap between the classes and predicts the class association of the test data samples based on which side of the gap they fall on. In this study, the Gaussian radial basis function was used as the kernel function for SVM (a kernel function defines how the data samples will be mapped into the new feature space called kernel space). Model parameters including *C* and *γ* were optimized using a grid search, where *C* is the soft margin parameter and *γ* is the free parameter of the RBF kernel.

In a leave-one-out cross-validation scheme, the k-NN classifier predicts the class association of a test point in the feature space based on the class which forms the majority of the points neighboring the point of interest, and based on the distance between those points and the point of interest. The latter two non-linear classifiers are favourable when the classes cannot be separated by a line and a large number of features is available. All classifier predictions were made using leave-one-patient-out cross-validation. In order to evaluate, statistically, how well a classifier could separate the two groups (good versus poor response patients), a Mann-Whitney test or a t-test (depending on the normality of the data) was performed on the estimated posterior probabilities of each group. The estimated posterior probability, *P*(*C*_*j*_|*x*), was defined as the estimated probability that the correct label, *C*, for *x*, is *C*_*j*_ and follows Bayes’ theorem^33^. In this study the statistical significance of the best classifier, *k-*NN classifier, was computed using the ratio of sum of the weights of the neighbors that belong to class *j*, i.e., *W*_*i*∈*Cj*_, to the sum of the weights of all neighbors, i.e., *W*_*i*_. The formulation for *P*(*C*_*j*_|*x*) is ref. 34


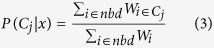


Where *nbd* represents the neighborhood (all data samples inside the neighborhood of size *k*). The weight represents the distance between each data sample and its neighbor.

## Results

A “patient-by-patient” clinical characteristics is presented in Supplementary Table 1. Patients were females with a mean age of 49 years (SD = 10) and a mean breast tumour size of 6.3 cm (SD = 3.2). The majority of patients (93%) had invasive ductal carcinoma (IDC), while 5% had infiltrating lobular carcinoma and 2% had other subtypes. In this study, 42 of the 56 patients demonstrated good response to NAC and the remaining 14 patients demonstrated poor response (less than a 30% diminishment in longest dimension) based on RECIST in addition to pathological criteria (see Methods). In the alternative classification scheme, 18 patients demonstrated complete response, and the remaining 38 patients were classified into the incomplete response group. The majority of the patients received anthracycline and taxane based treatment (48/56 or 86%), where 11 of these 48 patients (23%) also received the targeted drug Trastuzumab as part of their NAC (see Supplementary Table 1 for a complete list of individual treatments). A complete list of clinical/pathological responses and MP scores of individual patients is reported in Supplementary Table 2. A statistical summary of the clinical characteristics of study patients is presented in Supplementary Table 4. Patients were accrued from January 2009 to March 2013 and had a median follow-up of 2 years for this study.

A representative conventional ultrasound image (B-mode image) of a patient’s breast tumour, its corresponding core and margin ROIs, and a resulting SI parametric image are presented in Fig. 1. Supplementary Table 5 presents the classification performance of molecular markers of LABC tumours including ER, PR, and HER2 statuses, with values as positive or negative. Whereas ER demonstrated low sensitivity (55%) and high specificity (79%), PR and HER2 demonstrated an opposite trend (sensitivities of 95% and 60%, and specificities of 0% and 14%, respectively).

**Figure 2:**
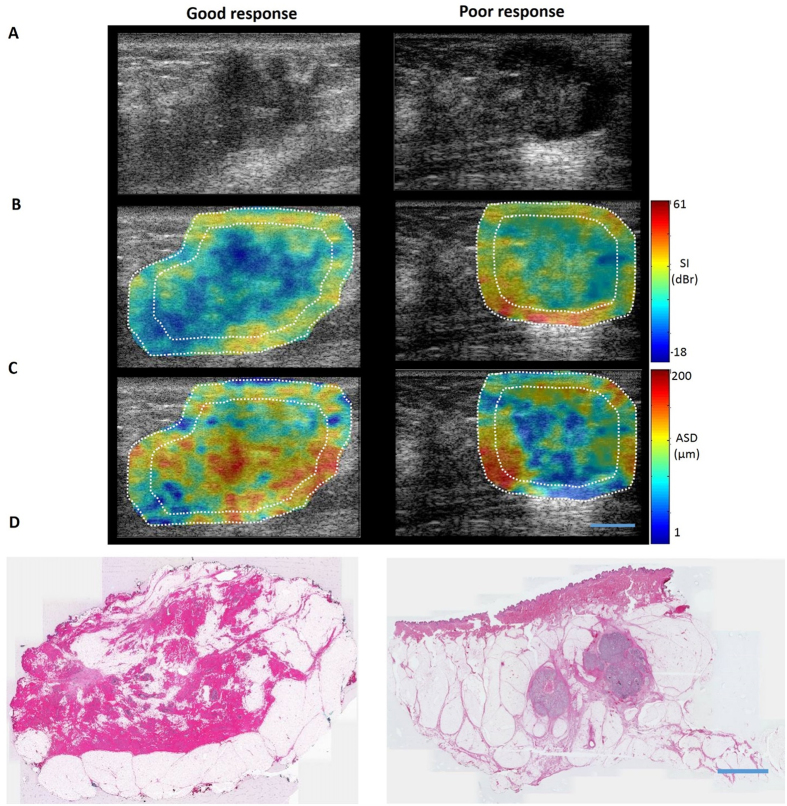
Comparison of a responding and a non-responding patient’s tumour. Original B-mode images (**A**), SI parametric images (**B**), ASD parametric images (**C**) with core and margin ROIs outlined in white, and H&E-stained post-surgical breast specimen (**D**) with pink indicating normal breast tissue, light pink indicating fibrosis, and purple indicating residual tumour tissue. Scale bars (US and histology): 1 cm.

Figure 2 displays a panel of images related to one representative good response patient and one representative poor response patient, including original B-mode images, parametric images of the best spectral parameter (SI), parametric images of the best backscatter-model-based parameter (ASD), and hematoxylin and eosin (H&E) -stained sections of the post-surgical breast specimen. The best parameters were determined based on their dominance in the optimal feature set presented in Table 1. As evident in Fig. 2A, a tumour in a B-mode image of a LABC patient’s breast can readily be identified as a hypo-intense mass surrounded by relatively hyper-intense fibroglandular tissue. As observed in Fig. 2B,C, parametric maps of SI and ASD hold further information about the tumour, with each ROI (core and margin) containing a unique textural pattern. The H&E section for the representative good response patient demonstrates only fibroglandular tissue (pink stain) and fibrosis (light pink stain) remaining in the tumour “bed”, whereas the H&E section for the poor response patient shows two distinct masses (purple stain) of residual tumour remaining after months of treatment.

**Figure 3:**
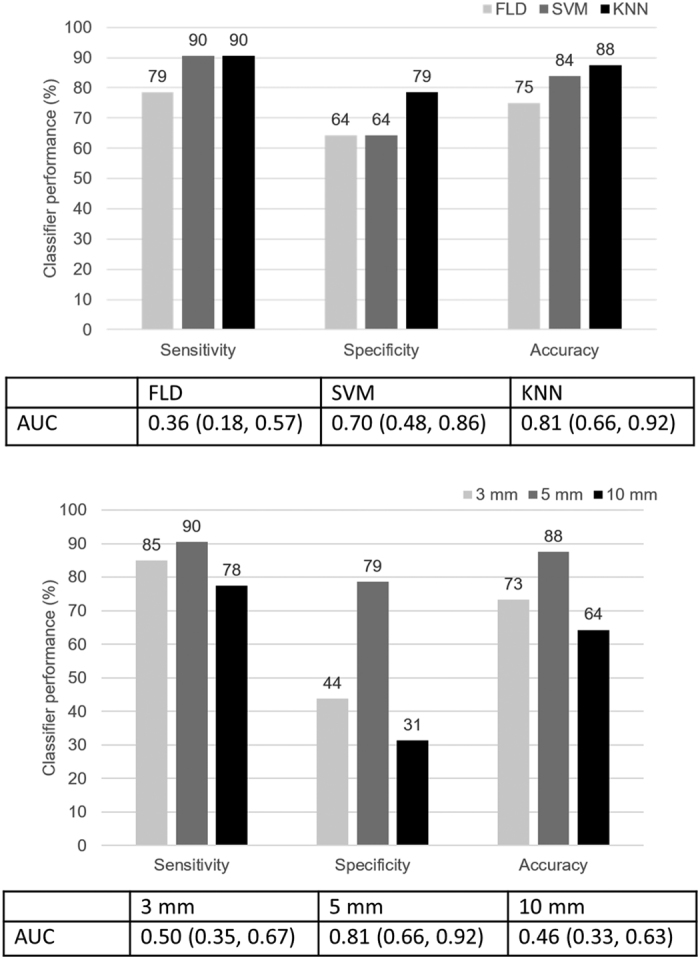
Patient response classification performance of different classifiers for a margin thickness of 5 mm (**A**) and for different margin thicknesses (**B**). The results in (**B**) are based on the classifier that performed the best for each margin thickness, which was the *k-*NN in in all three cases. The values in the parentheses beside the AUC represent the lower and upper bounds of the 95% confidence intervals of the AUC.

Figure 3A presents the classification performance results for good versus poor response classification obtained using three different classifiers (FLD, SVM, and *k-*NN) and using the optimal feature set obtained through sequential forward feature selection. Reported values are sensitivity, specificity, accuracy, and AUC. For AUC, 95% confidence bounds are reported, which were obtained through bootstrapping (1000 samples). As evident, the k-NN classifier provided the most favourable classification, with a sensitivity of 90%, a specificity of 79%, an accuracy of 88%, and an AUC of 0.81, for predicting response to chemotherapy in advance of it being administered. Figure 3B presents the classification performance results obtained for different margin thicknesses used to generate *ROI*_*margin*_, including 3, 5, and 10 mm thicknesses. Results suggested that 5 mm is the optimal margin thickness for characterizing a patient’s tumour responsiveness.

Table 1 presents the optimal QUS feature set and corresponding p-values obtained using the KNN classifier with 5 mm-thick margin ROI for good response versus poor response classification. ACE was determined to be the best discriminating parameter with a p-value = 0.019, whereas SI was the most dominant QUS parameter with four features appearing in the optimal feature set including 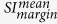, 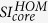, *SI*^*CMR*^, and *SI*^*CMCR*^ Other parameters also appeared in this feature set, including MBF (one feature), SS (two features), and ASD (one feature). Results suggest that the statistical and image quality features of spectral parameters provided more discriminatory information about the response characteristics of a tumour than backscatter model parameters such as ASD. Removal of ASD from the feature set resulted in a 2% decrease in classification accuracy (from 88% to 86%). Above all, a Mann-Whitney test on the posterior probabilities of the good response and poor response groups demonstrated highly statistically significant results (p < 0.001), as presented in the last row of Table 1, further demonstrating the effectiveness of the k-NN based multiparametric classifier in differentiating between the two response groups.

Table 2 compares response classification performances of the QUS model and the model combining QUS features with molecular markers for the two response grouping schemes. Results based on the best classifier (FLD, SVM, or k-NN) for each case are reported, which were the KNN classifier and 5- mm margin configuration for good versus poor response classification, and SVM and 5-mm margin for the complete versus incomplete response classification. For good versus poor response classification, the addition of molecular markers resulted in performance deterioration (accuracy decreased from 88% to 79%). For complete versus incomplete response classification, there was no noticeable difference between the QUS only and QUS + molecular features models in terms of classification accuracy (82% and 82%) and AUC (0.75 and 0.76).

**Figure 4:**
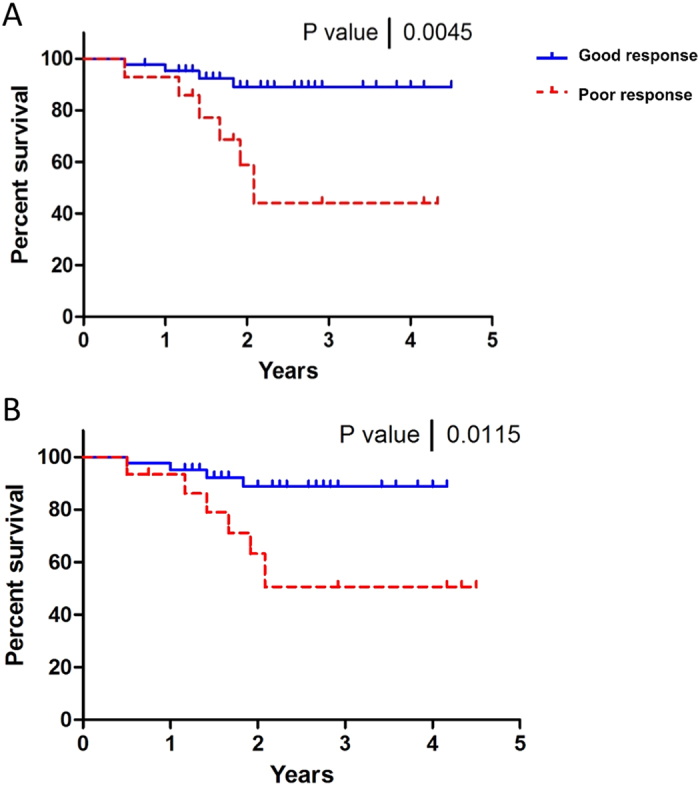
Five-year RFS curves for good response and poor response patients determined at the end of treatment based on clinical and pathological response (**A**), and based on QUS prediction prior to treatment initiation (**B**).

Figure 4 presents five-year RFS curves for good response and poor response patients calculated based on clinical response (RECIST and pathology criteria) (Fig. 4A) and those calculated based on QUS predictions (Fig. 4B, using the *k-*NN 5 mm-margin model). The RFS curve based on the QUS predictions was obtained in the same manner as the RFS curve based on clinical response, except that the classification of each patient as a survivor or non-survivor (recurrence or death) used for generating the curve was based on the QUS predictions. The QUS predictions were based on the k-NN classifier and optimal feature set reported in Table 1. As expected, good response patients had a higher survival rate (nearly 100%) compared to poor response patients based on their RECIST and pathological response, which was statistically significant (log-rank test, p-value < 0.005). In addition, QUS-based predictions demonstrated parallel trends with statistically significant differences in survival rates between the two groups (p-value = 0.01).

## Discussion

In this study, pre-treatment textural and image quality features of QUS parametric images of breast tumours and their margin zones were demonstrated to be predictive of chemotherapy responsiveness in LABC patients undergoing NAC treatment with an 88% classification accuracy. The classification results were verified with ultimate clinical responses of the patients determined based on MP criteria. Parameters of the predictive model including classifier type and tumour margin thickness were optimized for best response classification, yielding the k-NN classifier incorporating 5 mm- margin thickness. Furthermore, 5-year RFS curves of the LABC patients obtained using the predictive model followed closely with that obtained using clinical information comprised of RECIST-based response and post-surgical histopathology. MP scoring demonstrated equivalence with clinical-response criteria (RECIST) but provided additional information about microscopic pathological details. It was important to use MP scoring as tumour cellularity is taken into account in this methodology whereas RECIST-only criteria rely on anatomical measures which do not always accurately reflect biological tumour response.

According to findings of this study, the ACE parameter demonstrated the most statistically significant discrimination between tumours of good response and poor response patients. Other studies involving breast tissue characterization using QUS obtained similar results for differentiating benign from malignant breast features^35^,^36^ but without linkage to predicting treatment response. For instance, in D’Astous *et al*.^35^, measurements of frequency-dependent ultrasound attenuation and backscatter signal from *ex vivo* samples of breast tissues including fat, parenchyma, and infiltrating ductal carcinoma demonstrated that frequency-dependent attenuation was discriminative among those tissues. Similarly, Landini *et al*.^36^ demonstrated that invasive ductal carcinoma could be differentiated from fatty, fibrotic, and parenchymal tissues on the basis of its frequency-dependent attenuation. Just as textural features of QUS images were found to contribute to the differentiation of therapy responsive and non-responsive tumours here, the same features earlier provided a strong discrimination between low grade and medium-to-high grade breast tumours in Tadayyon *et al*.^16^.

The high accuracy of our QUS-based model in predicting tumour responsiveness (88%) can be attributed to the fact that the model includes multiple parameters, accounting for breast tissue composition and stiffness (ACE), microstructural size (i.e. SS and ASD), and microstructural and cellular organization of tissue (MBF and SI). The fact that the acoustic features of both the tumour core and its margin contributed to the classification suggests that the margin may account for the presence of microscopic “limbs” infiltrating from the primary tumour mass into surrounding normal tissue.

The poor performance of the FLD (75% accuracy) was not unexpected, as the task at hand is too complex for a linear algorithm such as FLD. The task at hand was differentiating breast tumours of mostly the same histological type (IDC) which are clinically and ultrasonically (B-mode) indistinguishable, but differing in their response characteristics to chemotherapy. The reason for the superior performance of the k-NN classifier over the SVM (4% higher accuracy) could be attributed to the fact that the k-NN classifier is easier to optimize since it generally requires only one parameter to be tuned, i.e. the number of neighbors parameter (*k*), whereas the SVM requires tuning of the cost (*C*) and kernel parameters (*γ*).

To date, investigators have mainly focused on development of non-invasive imaging biomarkers for monitoring response of breast cancer patients to cancer therapy^21^,^37^,^38^,^39^,^40^ and have obtained promising results. This would require a patient to endure one cycle or more of a potentially ineffective treatment before its ineffectiveness can be determined. The study here, however, identified QUS-based biomarkers with good accuracy (88%) which are predictive of response at baseline without requirement of treatment administration. This can potentially lead to substantial savings in time, costs, resources, and improve the short term effects (toxicity) and long term effects (survival) on the patients. The QUS-based predictive model proposed here is a tool for oncologists to help them determine the best course of treatment for LABC patients.

The poorer classification performances obtained for complete versus incomplete response classification compared to good versus poor response classification may be attributed to an abrupt transition between positive and negative response. In other words, patients who have nearly complete response with only small clusters of tumour cells remaining are classified as incomplete response, but may likely behave similarly to complete response patients. On the contrary, good versus poor response grouping provides a smoother transition between positive and negative response, which is based on a 30% tumour size reduction (and residual tumour cellularity).

A recent study by our group^23^ demonstrated favourable tumour response prediction using a multi-parameter QUS imaging approach similar to that proposed here, but using imaging data obtained during the treatment (weeks 1, 4, and 8) combined with pre-treatment data. The results indicated that combining pre-treatment data with intra-treatment data yielded a superior prediction accuracy compared to using intra-treatment data alone (70% compared to 60% at week 1, 80% compared to 77% at week 4, and 81% compared to 75% at week 8, respectively). These results motivated the current study - to investigate pre-treatment markers as potential stand-alone markers of tumour response. One of the limitations of the current approach is the manual segmentation of the ultrasound images. As Fig. 3B suggests, the classification performance is sensitive to the size of the tumour margin. Thus, a reproducible segmentation method is central to the performance consistency of the proposed QUS-based tumour response prediction system. In the future, we will investigate automated tumour segmentation methods and evaluate margin characteristics in order to overcome this limitation. We posit that margin thickness is important as this area contains microscopic disease extension or is an area that is affected by factors originating from gross tumour.

There is now mounting evidence suggesting that molecular subtypes of tumours play an important role in developing or inherently having drug resistance^41^. Correlating the acoustic properties of a tumour to its molecular subtype can facilitate understanding of drug resistant behaviors of tumours. A previous clinical study indicated that patients with HER2 positive breast cancer are more likely to achieve pathologic complete response to Taxol/Fluorouracil-Adriamycin-Cyclophosphamide (T/FAC) NAC^5^. However, the pathologically incomplete response group may consist of a mix of partially responsive, stable disease, and progressive disease patients, for which HER2 sensitivity was not investigated in that study. In the study here, the finding that HER2 was found to be highly sensitive (high positive response detection rate) but not specific (low negative response detection rate) to response was in agreement with the findings from a previous molecular biomarker study^5^ where a statistically significant correlation was found between HER2 status and complete response in breast cancer patients receiving T-FAC chemotherapy. Firstly, the latter results were demonstrated in a subgroup of patients who received a specific type of chemotherapy. Secondly, a statistically significant result based on a comparison of means of two groups does not necessarily correlate with classification sensitivity and specificity. Oncotype-Dx testing^42^ which measures risk recurrence in early breast cancer based on a score derived from a 21 gene - RT-PCR analysis has been used with adjuvant chemotherapy for predicting the need of chemotherapy but is not used as an upfront predictor of response to chemotherapy. Oncotype Dx scores were unavailable for the patients here as it remains outside of standard of care.

A more promising marker is Ki-67, which has been demonstrated to be predictive of breast tumour response to chemotherapy at baseline (p = 0.0001)^2^. In a future study, Ki-67 and QUS measurements will be made coincidently on breast cancer patient subjects prior to treatment initiation in order to assess the correlation between the two observations and to response. Tumour subtype was not significant in this study. Whether or not patients with ILC were included or excluded did not alter predictive results.

The method developed here may however be combined with monitoring of cell-death responses using quantitative ultrasound^21^. That study indicates importantly that image-based response to neoadjuvant chemotherapy correlates very well with long-term outcomes. It is not necessarily surprising then that information obtained before the start of chemotherapy using quantitative ultrasound would similarly correlate well to long-term patient outcomes as in this study.

In this study we propose that ultrasound-based microstructural tumour characteristics can also be used to predict tumour response to therapy. Here QUS and textural features, in principle, reflect tumour structural changes at a cellular level and also at a tissue organizational level. Such features are recognized to become more disorganized with aggressive tumours as reflected in the ultrasound features studied here. The method here provides a manner to non-invasively quantify tumour structural-based changes in tissue with links to patient clinical outcomes. The particular features which worked best appeared to be tumour core based parameters and also core-to-margin contrast ratio-based parameters. Higher-order model-based parameters such as the effective scatterer size and the acoustic-scatter concentration estimate also appeared in the feature set which best predicted for response. The use of texture parameters was critical in order to obtain the sensitivity and specificity reported in this study and it is posited that such texture-based features better reflect the structural heterogeneity that can develop in tumours.

The features in the optimal set here therefore likely span tumour structure in its core but also through the use of margin-to-tumour ratio features include its microscopic effects on surrounding tissue. In addition, the ultrasound-based predictions of response here were reflected in terms of 5-year patient survivals signifying real-life implications for such ultrasound-based predictions.

Other modalities have relied on similar mean-value and texture analyses including ultrasound spectroscopy, diffuse optical spectroscopy and MRI methods for therapy response monitoring^43^,^44^,^45^,^46^. In patients with triple negative disease pre-treatment, MRI-based kinetic maps have demonstrated positive results for chemotherapy response prediction in “triple-negative” breast tumours. A study utilizing diffuse optical spectroscopy also measured baseline tumour oxygen saturation and reported significantly higher values in patients with pathological complete response who received neoadjuvant treatment^43^. Results also strongly suggest that pre-treatment tumour heterogeneity can influence drug efficacy^44^. Texture analyses of dynamic contrast-enhanced MRI images have also been used^45^,^46^ to predict neoadjuvant chemotherapy response. Specifically, results have indicated significant differences in GLCM-texture features between chemotherapy responding and non-responders patients from pre-treatment data^46^.

The work in the study here had link to patient responses of chemotherapy in terms of tumour responsiveness and thus naturally has links to patients’ outcome in terms of survival. This type of testing *a priori* may aid in stratifying patients for adaptive chemotherapy whereby the treatment offered is customized based on tumour aggressiveness. For instance, if a patient is deemed to be responsive based on the QUS prediction, then standard first-line chemotherapy can be administered according to the patient’s tumour characteristics. Conversely, if a patient is deemed to be non-responsive, a more aggressive first-line chemotherapy regimen can be administered in order to maximize chances of positive response. This is already carried out in the adjuvant setting with testing through molecular assays such as Oncotype Dx. The research here offers the possibility to do so in a neoadjuvant setting using tumour structure characterization non-invasively through the use of ultrasound.

## Conclusion

In this study, a QUS-based multiparametric classifier incorporating texture and image quality features that account for tumour core and a 5 mm thick surrounding margin was demonstrated to be a sensitive (90%) and specific (79%) pre-treatment predictor of tumour response to therapy and 5-year RFS (p < 0.05). Pre-treatment image-based surrogates of response stand to personalize health care by minimizing drug toxicity and maximizing chances of long-term survival.

## Additional Information

**How to cite this article:** Tadayyon, H. *et al*. A priori Prediction of Neoadjuvant Chemotherapy Response and Survival in Breast Cancer Patients using Quantitative Ultrasound. *Sci. Rep.* **7**, 45733; doi: 10.1038/srep45733 (2017).

**Publisher's note:** Springer Nature remains neutral with regard to jurisdictional claims in published maps and institutional affiliations.

## Supplementary Material

Supplementary Information

## Figures and Tables

**Table 1:** Optimal feature set obtained through sequential forward feature selection using the *k*-NN classifier, for good response versus poor response classification.

Parameter	P-Value
ACE	0.019
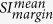	0.118
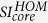	0.179
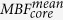	0.192
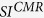	0.192
*ASD*^*CMCR*^	0.234
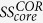	0.244
*SS*^*CMCR*^	0.303
*SI*^*CMCR*^	0.366
*k – NN model*	<0.001

**Table 2:** Comparison of response classification performance between QUS and QUS + molecular subtypes models.

Model	Se (%)	Sp (%)	Ac (%)	AUC
A. good response vs poor response grouping				
QUS (k-NN)	90	79	88	0.81 (0.66, 0.92)
QUS + Molecular subtypes (k-NN)	86	57	79	0.71 (0.55, 0.86)
B. complete response vs incomplete response grouping				
Model	**Se (%)**	**Sp (%)**	**Ac (%)**	**AUC**
QUS (SVM)	61	92	82	0.75 (0.54, 0.89)
QUS + Molecular subtypes (SVM)	67	89	82	0.76 (0.59, 0.88)

## References

[b1] Giordano S. H. Update on locally advanced breast cancer. Oncologist 8, 521–530 (2003). 14657530 10.1634/theoncologist.8-6-521

[b2] Chang J. . Apoptosis and proliferation as predictors of chemotherapy response in patients with breast carcinoma. Cancer 89, 2145–52 (2000). 11147583

[b3] Stoetzer O. J. . Prediction of response to neoadjuvant chemotherapy in breast cancer patients by circulating apoptotic biomarkers nucleosomes, DNAse, cytokeratin-18 fragments and survivin. Cancer Lett. 336, 140–148 (2013). 23612068 10.1016/j.canlet.2013.04.013

[b4] Lehner J., Stötzer O. J., Fersching D., Nagel D. & Holdenrieder S. Circulating plasma DNA and DNA integrity in breast cancer patients undergoing neoadjuvant chemotherapy. 425, 206–211 (2013). 10.1016/j.cca.2013.07.027 23916787

[b5] Andre F. . HER2 expression and efficacy of preoperative paclitaxel/FAC chemotherapy in breast cancer. Breast Cancer Res. Treat. 108, 183–190 (2008). 17468948 10.1007/s10549-007-9594-8

[b6] Jiang S. . Predicting Breast Tumor Response to Neoadjuvant Chemotherapy with Diffuse Optical Spectroscopic Tomography prior to Treatment. Clin. Cancer Res. 20, 6006–6015 (2014). 25294916 10.1158/1078-0432.CCR-14-1415 PMC4254218

[b7] Sadeghi-Naini A. . Imaging innovations for cancer therapy response monitoring. Imaging Med. 4, 311–327 (2012).

[b8] Schwarzenbach H. & Pantel K. Circulating DNA as biomarker in breast cancer. 17, 136 (2015). 10.1186/s13058-015-0645-5 PMC4599311 26453190

[b9] Coleman D. J. . A model for acoustic characterization of intraocular tumors. 26, 545–50 (1985). 3884539

[b10] Feleppa E. J. . Typing of prostate tissue by ultrasonic spectrum analysis. IEEE Trans. Ultrason. Ferroelectr. Freq. Control 43, 609–619 (1996).

[b11] Yang M., Krueger T. M., Miller J. G. & Holland M. R. Characterization of anisotropic myocardial backscatter using spectral slope, intercept and midband fit parameters. Ultrason. Imaging 29, 122–134 (2007). 17679326 10.1177/016173460702900204

[b12] Mamou J. . Three-dimensional high-frequency backscatter and envelope quantification of cancerous human lymph nodes. Ultrasound Med. Biol. 37, 345–57 (2011). 21316559 10.1016/j.ultrasmedbio.2010.11.020 PMC3062193

[b13] Insana M. F., Wagner R. F., Brown D. G. & Hall T. J. Describing small-scale structure in random media using pulse-echo ultrasound. J. Acoust. Soc. Am. 87, 179–92 (1990). 2299033 10.1121/1.399283 PMC2745727

[b14] Oelze M. L., O’Brien W. D., Blue J. P. & Zachary J. F. Differentiation and characterization of rat mammary fibroadenomas and 4T1 mouse carcinomas using quantitative ultrasound imaging. IEEE Trans. Med. Imaging 23, 764–771 (2004). 15191150 10.1109/tmi.2004.826953

[b15] Tadayyon H., Sadeghi-Naini A., Wirtzfeld L., Wright F. C. & Czarnota G. Quantitative ultrasound characterization of locally advanced breast cancer by estimation of its scatterer properties. Med. Phys. 41, 12903 (2014). 10.1118/1.4852875 24387530

[b16] Tadayyon H., Sadeghi-Naini A. & Czarnota G. J. Noninvasive characterization of locally advanced breast cancer using textural analysis of quantitative ultrasound parametric images. Transl. Oncol. 7, 759–67 (2014). 25500086 10.1016/j.tranon.2014.10.007 PMC4311023

[b17] Banihashemi B. . Ultrasound imaging of apoptosis in tumor response: novel preclinical monitoring of photodynamic therapy effects. Cancer Res. 68, 8590–6 (2008). 18922935 10.1158/0008-5472.CAN-08-0006

[b18] Vlad R. M., Brand S. & Giles A. Quantitative Ultrasound Characterization of Responses to Radiotherapy in Cancer Mouse Models. Clin. Cancer. Res. 15 **(6)**, 2067–2074 (2009). 19276277 10.1158/1078-0432.CCR-08-1970

[b19] Sadeghi-Naini A. . Conventional Frequency Ultrasonic Biomarkers of Cancer Treatment Response *In Vivo*. Transl. Oncol. 6, 234–243 (2013). 23761215 10.1593/tlo.12385 PMC3678128

[b20] Czarnota G. J. . Tumor radiation response enhancement by acoustical stimulation of the vasculature. Proc. Natl. Acad. Sci. USA 109, E2033–2041 (2012). 22778441 10.1073/pnas.1200053109 PMC3409730

[b21] Sadeghi-Naini A. . Early prediction of therapy responses and outcomes in breast cancer patients using quantitative ultrasound spectral texture. Oncotarget 5, 3497–3511 (2014). 24939867 10.18632/oncotarget.1950 PMC4116498

[b22] Sannachi L. . Non-invasive evaluation of breast cancer response to chemotherapy using quantitative ultrasonic backscatter parameters. Med. Image Anal, doi: 10.1016/j.media.2014.11.009 (2014). 25534283

[b23] Tadayyon H. . Quantitative ultrasound assessment of breast tumor response to chemotherapy using a multi-parameter approach. Oncotarget 7, 45094–45111 (2016). 27105515 10.18632/oncotarget.8862 PMC5216708

[b24] Wood W. C. Close/positive margins after breast-conserving therapy: Additional resection or no resection? The Breast 22, S115–S117 (2013). 24074771 10.1016/j.breast.2013.07.022

[b25] Eisenhauer E. A. . New response evaluation criteria in solid tumours: revised RECIST guideline (version 1.1). Eur. J. Cancer 45, 228–47 (2009). 19097774 10.1016/j.ejca.2008.10.026

[b26] Rajan R. . Change in tumor cellularity of breast carcinoma after neoadjuvant chemotherapy as a variable in the pathologic assessment of response. Cancer 100, 1365–73 (2004). 15042669 10.1002/cncr.20134

[b27] Symmans W. F. . Measurement of residual breast cancer burden to predict survival after neoadjuvant chemotherapy. 25, 4414–4422 (2007). 10.1200/JCO.2007.10.6823 17785706

[b28] Lizzi F. L., King D. L., Rorke M. C. & Others. Comparison of theoretical scattering results and ultrasonic data from clinical liver examinations. Ultrasound Med. Biol. 14, 377–385 (1988). 3051612 10.1016/0301-5629(88)90073-7

[b29] Labyed Y. & Bigelow T. a. A theoretical comparison of attenuation measurement techniques from backscattered ultrasound echoes. J. Acoust. Soc. Am. 129, 2316–24 (2011). 21476687 10.1121/1.3559677 PMC3087399

[b30] Haralick R. M., Shanmugam K. & Dinstein I. Textural Features for Image Classification. IEEE Trans. Syst. Man. Cybern. SMC-3, 610–621 (1973).

[b31] Gayou O. Influence of acquisition parameters on MV-CBCT image quality. 13, 3638 (2012). 10.1120/jacmp.v13i1.3638 PMC5716124 22231215

[b32] Jain A., Duin R. & Mao J. Statistical Pattern Recognition: A Review. IEEE Trans. Pattern Anal. Mach. Intell. 22, 4–37 (2000).

[b33] Lee P. Bayesian Statistics: An Introduction, 4th Edition. (Wiley, 2012).

[b34] Cover T. & Hart P. Nearest neighbor pattern classification. IEEE Trans. Inf. Theory 13, 21–27 (1967).

[b35] D’Astous F. T. & Foster F. S. Frequency dependence of ultrasound attenuation and backscatter in breast tissue. Ultrasound Med. Biol. 12 **(10)**, 795–808 (1986). 3541334 10.1016/0301-5629(86)90077-3

[b36] Landini L. & Sarnelli R. Evaluation of the attenuation coefficients in normal and pathological breast tissue. Med. Biol. Eng. Comput. 24, 243–247 (1986). 3528703 10.1007/BF02441619

[b37] Schelling M. . Positron emission tomography using [(18)F]Fluorodeoxyglucose for monitoring primary chemotherapy in breast cancer. 18, 1689–95 (2000). 10.1200/JCO.2000.18.8.1689 10764429

[b38] Sharma U., Danishad K. K. A., Seenu V. & Jagannathan N. R. Longitudinal study of the assessment by MRI and diffusion-weighted imaging of tumor response in patients with locally advanced breast cancer undergoing neoadjuvant chemotherapy. NMR Biomed. 22, 104–113 (2009). 18384182 10.1002/nbm.1245

[b39] Falou O. . Evaluation of neoadjuvant chemotherapy response in women with locally advanced breast cancer using ultrasound elastography. Transl. Oncol. 6, 17–24 (2013). 23418613 10.1593/tlo.12412 PMC3573650

[b40] Falou O. . Diffuse Optical Spectroscopy Evaluation of Treatment Response in Women with Locally Advanced Breast Cancer Receiving Neoadjuvant Chemotherapy. Transl. Oncol. 5, 238–246 (2012). 22937175 10.1593/tlo.11346 PMC3431033

[b41] Luqmani Y. A. Mechanisms of drug resistance in cancer chemotherapy. Med. Princ. Pract. 14 Suppl 1, 35–48 (2005). 10.1159/000086183 16103712

[b42] Hornberger J., Cosler L. & Lyman G. Economic analysis of targeting chemotherapy using a 21-gene RT-PCR assay in lymph-node-negative, estrogen-receptor-positive, early-stage breast cancer. 11, 313–24 (2005). 15898220

[b43] Ueda S. . Baseline tumor oxygen saturation correlates with a pathologic complete response in breast cancer patients undergoing neoadjuvant chemotherapy. Cancer Res. 72, 4318–4328 (2012). 22777823 10.1158/0008-5472.CAN-12-0056 PMC3609716

[b44] Golden D. I., Lipson J. A., Telli M. L., Ford J. M. & Rubin D. L. Dynamic contrast-enhanced MRI-based biomarkers of therapeutic response in triple-negative breast cancer. JAMIA 20, 1059–1066 (2013). 23785100 10.1136/amiajnl-2012-001460 PMC3822111

[b45] Teruel J. R. . Dynamic contrast-enhanced MRI texture analysis for pretreatment prediction of clinical and pathological response to neoadjuvant chemotherapy in patients with locally advanced breast cancer. NMR Biomed. 27, 887–896 (2014). 24840393 10.1002/nbm.3132

[b46] Ahmed A., Gibbs P., Pickles M. & Turnbull L. Texture analysis in assessment and prediction of chemotherapy response in breast cancer. J. Magn. Reson. Imaging 38, 89–101 (2013). 23238914 10.1002/jmri.23971

