# Supplementary material for: A priori Prediction of Neoadjuvant Chemotherapy Response and Survival in Breast Cancer Patients using Quantitative Ultrasound
Source: Sci Rep. 2017 Apr 12;7:45733. doi: 10.1038/srep45733 (PMC5388850; doi:10.1038/srep45733)
Supplement: Supplementary Information [file srep45733-s1.doc]

***- Supplementary Information***

***A priori Prediction of Neoadjuvant Chemotherapy Response and Survival in Breast Cancer Patients using Quantitative Ultrasound***

Hadi Tadayyon^1,2^, Lakshmanan Sannachi^1,2^, Mehrdad Gangeh^1,2^, Christina Kim^1,2^, Sonal Ghandi^4^, Maureen Trudeau^4^, Kathleen Pritchard^4^, William T. Tran^1,3^, Elzbieta Slodkowska^5^, Ali Sadeghi-Naini^1,6^, and Gregory Czarnota*^1-3,6^

^1^Physical Sciences, Sunnybrook Research Institute, Sunnybrook Health Sciences Centre, Toronto, ON, Canada.

^2^Department of Medical Biophysics, Faculty of Medicine, University of Toronto, Toronto, ON, Canada.

^3^Department of Radiation Oncology, Odette Cancer Centre, Sunnybrook Health Sciences Centre, Toronto, ON, Canada.

^4^Division of Medical Oncology, Department of Medicine, Sunnybrook Health Sciences Centre, Toronto, ON, Canada.

^5^Department of Anatomic Pathology, Sunnybrook Health Sciences Centre,

Toronto, ON, Canada

^6^Department of Radiation Oncology, Faculty of Medicine, University of Toronto, Toronto, ON, Canada.

**Supplementary Table 1.** Patient clinical characteristics, tumor dimensions and pathology, and administered treatment regiments. [Drug]* = maintenance drug given after surgery (not part of patient’s NAC). Asterisks beside a patient indicates that the patient was non-LABC, however, included in the study since they received NAC due to their invasive pathology, and/or nodal involvement. IDC = invasive ductal carcinoma, ILC = Invasive lobular carcinoma, IMC = invasive micropapillary carcinoma, ER = estrogen receptor expression, PR = progesterone receptor expression, HER2 = human epidermal growth factor receptor 2, A = anthracycline, T = taxane, Tr. = Trastuzumab, FEC = Fluorouracil-Epirubicin-Cyclophosphamide then Docetaxel, AC = Adriamycin/cyclophosphamide.

| **Patient No.** | **Age** | **Pre-tx tumor size (AP × ML × SI cm)** | **Path** | **ER** | **PR** | **HER2** | **Lymph Nodes Disease** | **Treatment regimen** | **Drug type** |
| --- | --- | --- | --- | --- | --- | --- | --- | --- | --- |
| 1 | 55 | 5.4 × 5 × 2.3 | IDC | - | + | + | + | FEC + Taxol, Trastuzumab | A/T + Tr. |
| 2 | 53 | 7.4 × 7.0 | IDC with mucineous features | + | + | - | + | Epirubicin + Docetaxel | A/T |
| 3 | 41 | 5.3 × 4.4 × 4.7 | IDC | + | + | + | + | Docetaxel, Carboplatin, Trastuzumab | T + Tr. |
| 4 | 65 | 10.0 × 10.0 | IDC | - | - | - | + | AC + Docetaxel | A/T |
| 5 | 50 | 2.2 | IDC | + | + | + | + | AC + Docetaxel, Trastuzumab* | A/T |
| 6 | 33 | 5.4 × 5.0 × 8.0 | IDC | + | + | + | - | AC + Docetaxel, Taxol, Trastuzumab | A/T + Tr. |
| 7 | 48 | 4.9 × 4.9 × 4.1 | IDC | + | + | - | + | AC + Docetaxel | A/T |
| 8 | 36 | 4.4 × 3.9 × 5.8 | IDC | + | + | - | + | AC + Taxol | A/T |
| 9 | 40 | 4.4 × 3.4 | IDC | - | - | - | + | AC + Taxol | A/T |
| 10 | 62 | 12.0 × 14.0 | IDC | - | - | - | + | FEC + Taxol | A/T |
| 11 | 59 | 6.0 × 2.3 × 4.3 | IDC | - | - | - | + | AC + Taxol | A/T |
| 12 | 50 | 13.0 × 11.0 | IDC | - | - | - | + | AC + Taxol | A/T |
| 13 | 49 | 7.1 × 5.5 × 8.9 | IDC | - | - | + | + | Docetaxel, Trastuzumab | T + Tr. |
| 14 | 46 | 15.0 | IDC | - | - | - | + | AC + Cisplatin | A |
| 15 | 40 | 3.0 × 2.4 × 3.0 | IDC | + | + | + | + | AC + Taxol, Trastuzumab | A/T + Tr. |
| 16 | 56 | 2.4 × 2.7 × 3.2 | IDC | - | - | + | + | AC + Taxol, Trastuzumab | A/T + Tr. |
| 17 | 49 | 2.4 by 2.8 × 1.4  (clinical:  4 × 5) | IDC | - | - | + | - | AC-Taxol + Trastuzumab | A/T + Tr. |
| 18 | 47 | 5.2 × 4.0 × 4.0 | IDC | + | + | - | + | FEC-D | A/T |
| 19 | 52 | 4.1 × 3.0 × 2.5 | IDC | + | + | - | + | AC + Docetaxel, Taxol | A/T |
| 20 | 44 | 9.9 × 4.5 × 9.7 | IDC | + | + | + | + | AC + Taxol, Trastuzumab | A/T + Tr. |
| 21 | 38 | 9.0 × 6.6 × 6.0 | IDC | + | + | - | + | AC + Taxol | A/T |
| 22* | 58 | 1.9 × 1.4 × 1.6  (clinical: 3 × 3.5) | IDC with basal like features | - | - | - | - | AC + Taxol | A/T |
| 23 | 35 | 5.9 | IDC | - | - | - | + | AC-Taxol | A/T |
| 24 | 38 | 8.0 × 8.0 | IDC | - | - | + | - | Dose-Dense AC + Taxol, Trastuzumab | A/T + Tr. |
| 25 | 47 | 8.0 × 10.0 | IDC | + | + | - | - | Dose-Dense AC + Taxol | A/T |
| 26 | 57 | 7.9 × 4.1 × 5.5 | IDC | - | - | - | + | Dose-Dense AC + Taxol | A/T |
| 27 | 47 | 6.3 × 4.1 × 7.4 | IDC | - | - | + | + | Dose-Dense AC + Taxol, Trastuzumab* | A/T |
| 28 | 55 | 6.6 × 12.8 × 6.8 | IDC | + | + | - | + | AC + Taxol | A/T |
| 29 | 32 | 6.0x7.0x3.0 | IMC | + | + | + | + | AC + Taxol + Trastuzumab* | A/T |
| 30* | 38 | 2.3 × 2.5 × 2.5 & 1.0 × 1.0 × 0.7 | IDC | - | - | - | - | AC + Taxol | A/T |
| 31 | 45 | 6.5 × 5.0 | IDC | + | + | + | + | AC+Taxol + Trastuzumab | A/T + Tr. |
| 32 | 55 | 10.0 × 5.0 × 10.5 | IDC | - | - | - | + | Dose Dense AC + Taxol | A/T |
| 33 | 59 | 8.0 × 5.7 x3.0 | IDC | + | + | + | + | FEC-D, Trastuzumab | A/T + Tr. |
| 34 | 37 | 2.5 × 2.0 | IDC | + | + | - | + | Dose Dense AC + Taxol | A/T |
| 35 | 50 | 9.0 × 7.0 × 3.0 | IDC | + | + | - | + | AC + Taxol | A/T |
| 36 | 54 | 2.3 | IDC | + | - | - | + | Docetaxel + cyclophosphamide | T |
| 37 | 55 | 1.6 x1.2 | ILC | + | + | - | + | FEC-D | A/T |
| 38 | 50 | 7.3 × 2.5 × 7.3 | IDC | - | - | - | + | FEC-D | A/T |
| 39 | 55 | 3.3 × 3.4 × 3.4  (clinical: 5) | IDC | - | - | - | - | Docetaxel + cyclophosphamide | T |
| 40 | 44 | 3.0x3.5x1.5 | IDC with prominent lymphoid stroma | - | - | - | + | FEC-D | A/T |
| 41 | 60 | 8.7 × 9.0 × 5.2 | ILC | + | - | - | + | FEC-D | A/T |
| 42 | 64 | 6.4 × 3.2 × 8.7 | ILC | + | + | - | + | FEC-D | A/T |
| 43* | 67 | 3.2 × 8.7 | IDC | - | - | - | - | FEC-D | A/T |
| 44* | 52 | 2.6 × 1.2 × 1.6 | IDC | - | - | - | - | FEC-D | A/T |
| 45 | 47 | whole breast replaced by tumour | IDC | - | - | - | + | FEC-D | A/T |
| 46 | 56 | 10.0 × 10.0 | IDC | + | + | + | + | Taxol + Trastuzumab | T + Tr. |
| 47 | 45 | 2.3 × 2.0 | IDC | + | + | + | + | FEC-D. | A/T |
| 48 | 59 | 4.9 × 2.1 × 1.4 | IDC | + | + | - | + | FEC-D | A/T |
| 49 | 66 | 3.5 × 5.2 × 2.1 | IDC | + | + | + | + | Docetaxel + cyclophosphamide | T |
| 50 | 49 | 1.8 × 2.1 × 2.1 | IDC | + | + | + | + | Dose Dense AC + Taxol + Trastuzumab | A/T + Tr. |
| 51 | 39 | 6.3 | IDC | + | + | - | + | FEC-D | A/T |
| 52 | 62 | 4.4 × 6.3 × 3.3 | IDC | - | - | - | - | Dose Dense AC + Taxol | A/T |
| 53 | 58 | 5.2 × 5.2 × 4.4 | IDC | + | + | + | + | Dose Dense AC + Taxol + Trastuzumab | A/T + Tr. |
| 54 | 58 | 2.3 × 4 × 2.3;1.6 × 1.8 × 1.6 | invasive mammary carcinoma | - | - | + | + | Docetaxel + Carboplatin + Trastuzumab | T |
| 55 | 45 | 2.7 × 3.2 × 2.0 | IDC | + | + | - | + | AC + Taxol | A/T |
| 56 | 29 | 4.2 × 2.9 × 2.7 | IDC | + | + | - | + | Dose Dense AC + Taxol | A/T |

**Supplementary Table 2.** Treatment outcomes for individual patients. MP=Miller-Payne Criteria, CR = complete response, PR = partial response, SD = stable disease, PD = progressive disease, NA = not applicable. In column 5, any response other than CR is classified as IR (incomplete response) in the alternative response grouping scheme.

| **Patient No.** | **Residual tumour bed size  (AP × ML × SI )** | **Clinical and pathological response** | **MP Score** | **RECIST-based response** |
| --- | --- | --- | --- | --- |
| 1 | No Residual Disease | good | 5 | CR |
| 2 | 7.0 × 5.0 × 3.0 | good | 3 | NA |
| 3 | 2.7 × 2.5 × 2.4 | poor | 2 | SD |
| 4 | 1.6 × 0.8 × 0.5 | good | 3 | PR |
| 5 | No Residual Disease | good | 5 | CR |
| 6 | No Residual Disease | good | 5 | CR |
| 7 | 1.4 × 1.0 × 1.0 | good | 3 | PR |
| 8 | 11.4 | poor | 1 | PD |
| 9 | No Residual Disease | good | 5 | CR |
| 10 | No Residual Disease | good | 5 | CR |
| 11 | 2.6 × 2.5 × 2.5 | good | 3 | PR |
| 12 | 4.0 | good | NA | PR |
| 13 | No Residual Disease | good | 5 | CR |
| 14 | No Residual Disease | good | 5 | CR |
| 15 | No Residual Disease | good | 5 | CR |
| 16 | 0.2 × 0.2 | good | 4 | PR |
| 17 | 1.4 × 2.4 × 1.4 | good | 4 | PR |
| 18 | 6.5 | good | 3 | NA |
| 19 | No Residual Disease | good | 5 | CR |
| 20 | 2.0 × 1.0 × 1.0 & 1.6 × 1.0 × 0.5 | good | 3 | PR |
| 21 | 2.9 × 2.0 × 1.5 & 2.0 × 1.5 × 1.0 | good | 3 | PR |
| 22 | 0.15 | good | 4 | PR |
| 23 | No Residual Disease | good | 5 | CR |
| 24 | No Residual Disease | good | 5 | CR |
| 25 | 12.5 × 4.5 × 3.5 | poor | NA | SD |
| 26 | No Residual Disease | good | 5 | CR |
| 27 | No Residual Disease | good | 5 | CR |
| 28 | 17.0 | poor | 1 | NA |
| 29 | 0.6 | good | 4 | PR |
| 30 | 2.8 × 3.0 × 2.3 & 1.5 × 1.6 × 1.1 | poor | 1 | PD |
| 31 | 2.8 + 2.0 | poor | 2 | SD |
| 32 | 2 foci (0.3+0.5) | good | 3 | PR |
| 33 | No Residual Disease | good | 5 | CR |
| 34 | 2.2 × 1.5 × 1.1 | poor | 2 | SD |
| 35 | 1.2 | good | 4 | PR |
| 36 | 5.5 | good | 3 | NA |
| 37 | 1.2 × 0.9 × 0.7 | poor | 2 | SD |
| 38 | 2.1 | good | 3 | PR |
| 39 | 1.8 | good | 3 | PR |
| 40 | No Residual Disease | good | 5 | CR |
| 41 | 8.0 × 5.0 × 4.5 & 3.0 × 2.5 × 1.7 | poor | 1 | PD |
| 42 | 19.0 | poor | 1 | PD |
| 43 | 3.2 × 3.0 × 1.8 | good | 3 | PR |
| 44 | 2.5 × 0.4 × 0.4 | good | 4 | NA |
| 45 | 4.5 × 3.1 × 2.9 | good | 3 | PR |
| 46 | 8.4 × 5.1 × 2.8 | good | 3 | NA |
| 47 | No Residual Disease | good | 5 | CR |
| 48 | 2.8 × 2.5 × 1.5 | good | 3 | PR |
| 49 | 4.0 × 3.0 | poor | 2 | SD |
| 50 | No Residual Disease | good | 5 | CR |
| 51 | 1.7.0×1.5.0×1.0 | good | 3 | PR |
| 52 | 12.6 × 6.0 × 3.0 | poor | 1 | PD |
| 53 | 3.4 | good | 3 | PR |
| 54 | No Residual Disease | good | 5 | CR |
| 55 | 3.0 | poor | 2 | SD |
| 56 | 4.0 | poor | 1 | SD |

**Supplementary Table 3**

QUS features which were extracted from the parametric images as potential prognostic biomarkers, categorized by the type of analysis and regions considered.

|  | **1^st^ Order Statistics** | | **2d Order Statistics** | **Image Quality** | |
| --- | --- | --- | --- | --- | --- |
| **Regions Considered** | **ROI_core_** | **ROI_margin_** | **ROI_core_** | **ROI_core_** | **ROI_margin_** |
| **Features**  **Computed** | Mean | | CON | CMR | |
|  |  | | COR | CMCR | |
|  |  | | ENE |  | |
|  |  | | HOM |  | |

**Supplementary Table 4: Summary of patient characteristics. IDC = invasive ductal carcinoma, ILC = Invasive lobular carcinoma.**

| **Feature** | **Value** | |
| --- | --- | --- |
| Age (years) | 49 ± 10 | |
| Pre-treatment tumour size (cm) | 6.3 ± 3.2 | |
|  | **n** | **%** |
| **Tumour Subtype** |  |  |
| IDC | 52 | 93 |
| ILC | 3 | 5 |
| Other | 1 | 2 |
| Good response | 42 | 75 |
| Size change (%) | 68 ± 47 | |
| Poor response | 14 | 25 |
| Size change (%) | -16 ± 57 | |

**Supplementary Table 5.**  Classification performance of molecular markers of response using the KNN classifier. The reported values in brackets beside the AUC are the lower and upper bounds of the 95% confidence of the AUC.

| **Parameter** | **Se (%)** | **Sp (%)** | **Ac (%)** | **AUC** |
| --- | --- | --- | --- | --- |
| **ER** | 55 | 79 | 61 | 0.67 (0.51, 0.78) |
| **PR** | 95 | 0 | 71 | 0.48 (0.42, 0.50) |
| **HER2** | 60 | 14 | 48 | 0.37 (0.27, 0.52) |
